# GTAG- and CGTC-tagged palindromic DNA repeats in prokaryotes

**DOI:** 10.1186/1471-2164-14-522

**Published:** 2013-07-31

**Authors:** Pier Paolo Di Nocera, Eliana De Gregorio, Francesco Rocco

**Affiliations:** 1 Dipartimento di Medicina Molecolare e Biotecnologie Mediche, Università Federico II, Napoli, Via S. Pansini 5 80131, Naples, Italy

**Keywords:** Palindromic sequences, Repeated DNA families, RNA hairpins, Transposases, Mobile DNA, Intragenic DNA elements

## Abstract

**Background:**

REPs (Repetitive Extragenic Palindromes) are small (20–40 bp) palindromic repeats found in high copies in some prokaryotic genomes, hypothesized to play a role in DNA supercoiling, transcription termination, mRNA stabilization.

**Results:**

We have monitored a large number of REP elements in prokaryotic genomes, and found that most can be sorted into two large DNA super-families, as they feature at one end unpaired motifs fitting either the GTAG or the CGTC consensus. Tagged REPs have been identified in >80 species in 8 different phyla. GTAG and CGTC repeats reside predominantly in microorganisms of the gamma and alpha division of Proteobacteria, respectively. However, the identification of members of both super- families in deeper branching phyla such Cyanobacteria and Planctomycetes supports the notion that REPs are old components of the bacterial chromosome. On the basis of sequence content and overall structure, GTAG and CGTC repeats have been assigned to 24 and 4 families, respectively. Of these, some are species-specific, others reside in multiple species, and several organisms contain different REP types. In many families, most units are close to each other in opposite orientation, and may potentially fold into larger secondary structures. In different REP-rich genomes the repeats are predominantly located between unidirectionally and convergently transcribed ORFs. REPs are predominantly located downstream from coding regions, and many are plausibly transcribed and function as RNA elements. REPs located inside genes have been identified in several species. Many lie within replication and global genome repair genes. It has been hypothesized that GTAG REPs are miniature transposons mobilized by specific transposases known as RAYTs (REP associated tyrosine transposases). RAYT genes are flanked either by GTAG repeats or by long terminal inverted repeats (TIRs) unrelated to GTAG repeats. Moderately abundant families of TIRs have been identified in multiple species.

**Conclusions:**

CGTC REPs apparently lack a dedicated transposase. Future work will clarify whether these elements may be mobilized by RAYTs or other transposases, and assess if de-novo formation of either GTAG or CGTC repeats type still occurs.

## Background

Repetitive sequences occur in large quantities in eukaryotic cells, but they also constitute a significant fraction of the DNA of many prokaryotic genomes. According to the sizes, prokaryotic DNA repeats may be broadly sorted into two main groups. Large repeats are mostly represented by IS (Insertion Sequences). IS measure 0.8-2 kb, feature terminal inverted repeats (TIRs) and encode endonucleases which interact with TIRs promoting IS mobilization [1,2]. Small repeats vary in size from 20 to 300 bp, have different structures and can be sorted into a few distinct classes [3]. One is represented by tandemly arranged repeats called CRISPRs (Clustered Regularly Interspaced Short Palindromic Repeats). CRISPRs measure 24 to 48 bp, and are located at one or more loci in several prokaryotic genomes, separated by regularly sized, non-repetitive sequences, which originate from the processing of plasmid and/or bacteriophage DNA, mediated by CRISPR-associated proteins. Spacer sequences serve as a 'memory' of past exposures to foreign DNA, and are used to recognize and silence exogenous genetic elements in a manner analogous to RNAi in eukaryotic organisms [4]. CRISPRs usually show some dyad symmetry but are not truly palindromic, and thus structurally differ from the elements called REPs (Repetitive Extragenic Palindromes). REPs are 20–40 bp long palindromic repeats, early described as an abundant component of the *Escherichia coli* genome (reviewed in [5]), and later shown to represent a significant fraction of the extragenic space of many prokaryotic genomes [6-9]. REPs are found as single units, but also close to each other, and pairs as larger clusters of REPs are referred to as BIME (Bacterial Interspersed Mosaic Elements). REPs and BIMEs have been hypothesized to play a role in processes as diverse as DNA supercoiling, transcription termination, mRNA stabilization [10,11]. Moreover, REPs can affect genome plasticity, by functioning as targets for insertion of IS sequences in Pseudomonas, Neisseria and Sinorhizobium Genus [12]. REP-like elements known as RPEs (Repetitive Palindromic Elements) were identified in the genome of the obligate intracellular bacterium *R. conorii*, and many found surprisingly inserted in-frame within open reading frames which likely encode functional proteins [13,14]. The third group of small prokaryotic DNA repeats is constituted by MITEs (Miniature Inverted-repeat Transposable Elements), 70–300 bp elements which resemble degenerated ISs, as they feature 15–30 bp TIRs, but have no coding capacity. The group of bacterial MITEs includes RUP elements in *Streptococcus pneumoniae*[15], NEMIS elements in *Neisseria meningitidis*[16,17], Bcr1 elements in *Bacillus cereus*[18], ERIC and YPAl elements in *Yersinia enterocolitica*[19,20], Nezha elements in Cyanobacteria [21], EFAR elements in *Enterococci*[22]. MITEs are often inserted next to coding sequences, are transcribed and influence the expression of neighboring genes by folding into robust secondary structures, which can either stabilize the mRNA, or alternatively accelerate its degradation [23]. MITEs can be mobilized by transposases recognizing their TIRs [15,16,24]. REPs may be miniature non-autonomous mobile DNA elements as well, since they are often associated to genes encoding transposases of the IS200/IS605 family, accordingly called RAYTs (REP-associated tyrosine transposases; ref. [25]).

REPs characteristically terminate at one end with the tetranucleotide GTAG [9,25,26]. Intriguingly, we found that *R. conorii* RPE sequences terminate at one end with the tetranucleotide CGTC. We have identified in prokaryotic genomes several families of short palindromic repeats alternatively tagged at one end either by GTAG or CGTC tetranucleotides. Multiple families of either or both repeat types reside in some microorganisms. Structure, genomic organization, chromosomal arrangement, degree of inter- and intraspecies variation, pattern of interspersion with coding regions of all these sequences are reported. The role played by specific transposases in the formation and maintenance of the various repeats is discussed. In several species, RAYT genes are not flanked by REPs, but rather by long TIRs. In some of them, moderately abundant families of TIR repeats have been identified.

## Results

Short SLSs tagged at one end by the tetranucleotide GTAG or CGTC mark the genome of several microorganisms. According to their branching patterns in the 16S rRNA trees, bacteria are divided into main phyla. GTAG repeats have been identified in microorganisms belonging to the Proteobacteria, Cyanobacteria, and Chloroflexi phyla, and the PVC (Planctomycetes, Verrucomicrobia and Chlamydiales; see ref. [27]) superphylum. GTAG repeats were found in all divisions (alpha to epsilon) of Proteobacteria, but predominate in bacteria of the late-branching [28] gamma division. Cyanobacteria occur as unicellular and multicellular microorganisms [29], and GTAG elements were found in both cell types. CGTC repeats were identified in microorganisms belonging to 5 phyla: Proteobacteria, Chlorobi, Bacteroidetes, Spirochaetes, Thermotogae. In contrast to GTAG repeats, CGTC repeats predominate in Proteobacteria of the alpha division. Most reside in free-living organisms, but some have been identified in obligate intracellular bacteria, such *Wolbachia* and *Rickettsiae*. CGTC and GTAG repeats coexist in *Neisseriae*, *Bradyrhizobium*, *Rhodopseudomonas palustris*, *Sulfurovum* sp. NB37-1, and *Coxiella burnetii*. This bacterium substantially differs from typical obligate intracellular bacteria because having a relatively large genome and most metabolic pathways intact, and may indeed be considered a facultative intracellular bacterium [30].

Features and properties of the identified GTAG and CGTC repeat families are described below.

### GTAG families

**Figure 1:**
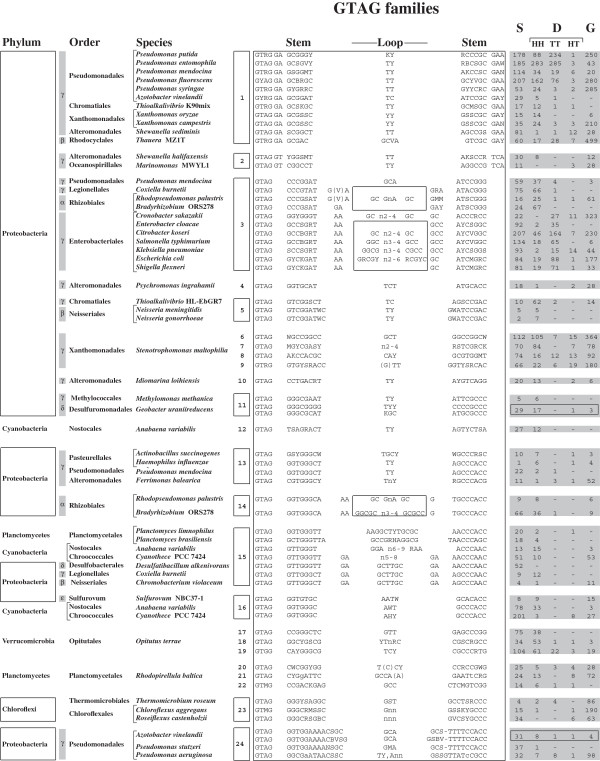
**Families of GTAG repeats.** The consensus sequences of GTAG-1 to GTAG-24 repeat families are reported. Families present in more than one species are boxed. Only the species, order and phyla are indicated (alpha to epsilon refer to Proteobacteria subdivisions). The complete names of the strains analyzed, and the NCBI accession numbers of the genomes are in Additional file 6. Loop sequences common to GTAG-3 and GTAG-14 elements from different species are boxed. Residues not present in all family members are in parentheses. Complementary nucleotide changes are indicated according to the NC-IUB codes (R=A,G; Y=C,T; K= G,T; M=A,C; S=G,C; W=A,T; B=C,G,T; H=A,C,T; V=A,C,G). Non complementary stem residues are in lowercase letters. Gray numbers to the right refer to single elements (S), dimers (D: HH, TT or HT types; see text) or grouped elements (G) in each family. Elements featuring alternative stem and loop sequences in *G. uraniireducens* GTA-11 and *A. vinelandii* GTAG-24 have been separately reported, but counted together (boxed gray numbers).

GTAG families have been sorted into 24 families (Figure 1). The classification takes into account changes of the stems, in terms of length (6–13 bp) and base composition, as changes of the loops, which measure 2–3 bp in many families, but vary in length among members of some families (Figure 1). Some GTAG families are restricted to one species only, others reside in multiple species of the same genus or order, as in evolutionary distant microorganisms. Repeats conserved in a genus have been analyzed in detail in strains of one or more species selected in the past for similar studies by other investigators. REPs identified in *Escherichia*[5] and *Pseudomonas*[6,7] genomes correspond to some of the GTAG-3 and GTAG-1 families listed in Figure 1, respectively. GTAG families 6 to 9 include all the *S. maltophilia* repeats previously called SMAGs [9]. Different REP families coexist also in *A. vinelandii*, *C. burnetii*, *R. palustris*, *Bradyrhizobium* sp. ORS278, *A. variabilis*, *Cyanothece* sp. PCC 7424, *O. terrae*, *R. baltica*. In contrast, different REPs reside in the two sequenced isolates of the *Thioalkalivibrio* genus *Thioalkalivibrio* sp. K90mix (GTAG-1 elements) and *Thioalkalivibrio* sp HL-EbGR7 (GTAG-5 elements).

Elements in Figure 1 are diagrammed in a modular fashion, to facilitate data presentation. In complex stem-loop structures, as those featured by *E. coli* REPs, some complementary bases are viewed as part of the loop region, rather than of bulged stems. Elements assigned to different families have different stem or loop sequences, or both. The terminal GTAG motif, conserved in >90% of the members of most repeat families, is variously degenerated in second and third position (GYAG, GYRG, GTRG, GTMG) in some families, and mutated to GTGG in the majority of *O. terrae* GTAG-20 elements. Most stems measure 6–9 bp. GTAG-1 repeats *in Thauera* sp. MZ1T have shorter stems (5 bp), all GTAG-24 repeats long (12–13 bp) stems. In the latter, complementarity is interrupted by mismatches in *P. aeruginosa* elements (unpaired GA residues in fifth position in all), 1 bp bulges due to the presence/absence of residues in tenth position in GTAG-24 repeats in other species.

Most families can be subdivided into sub-families made by units which feature alternative complementary stem residues, as denoted by the NC-IUB code in Figure 1. GT pairing of stem residues was often observed, suggesting that many GTAG repeats may be transcribed and function as RNA elements. GTAG-1 and GTAG-2 markedly differ from all other repeats as they feature dinucleotides not involved in base pairing between the SLS region and the GTAG terminus, and conserved 3 bp motifs at the opposite side (Figure 1).

Loops come in a few main formats. Most loops are very short, and many fit the consensus TY or CMA. Minimal size loops (2–4 bp) are compatible with the formation of RNA hairpins [31]. Some loops, in contrast, have a complex structure. In all GTAG-3 elements but those found in *P. mendocina*, non complementary di- and trinucleotides separate stem and loop sequences. The simplest loops are featured by *C. burnetii*, *C. sakazaki* and Rhizobial elements, and consist of 2–4 bp regions flanked by GC residues. In other GTAG-3 families, loops with complementary GC/GC, GGC/GCC, and GRCG/CGYC termini coexist (see boxed sequences in Figure 1). The inner regions of the GRCG/CGYC loops are self complementary, and up to 6–7 bp paired regions can be formed. The relative abundance of loop types varies among GTAG-3 elements in different species. Long loops predominate among *E. coli* and *S. flexneri* elements, but are missing in *E. cloacae*. In contrast, units with GGC/GCC loops are missing in *E. coli* and *S. flexneri*, but represent more than 50% of the GTAG-3 elements in *K. pneumoniae*.

GTAG-14 repeats feature loops exhibiting a similar organization, and two and three major loop variants with different GC-rich termini were identified in *R. palustris* and *Bradyrhizobium*, respectively (Figure 1). The inner region of the GCGG/CCGC type loops, which have been found only in *Bradyrhizobium* elements, is made by complementary residues, and may measure up to 27 bp. Large loops (9–15 bp) are a feature of GTAG-15 elements. These loops are partly related in sequence and have the same termini of GTAG-3 and GTGA-14 repeat loops, but complementary bases are missing.

GTAG repeats may be found as single units, but many are associated and form characteristic structures. In several families, repeats are predominantly associated as dimers. Elements are next to each other (1–5 bp distance) in some dimers, but are located 20–100 bp apart in most. The relative orientation of partners determines the formation of three types of dimers. Dimers carrying GTAG termini outside or inside are referred as HH (head-head), and TT (tail-tail), respectively, those made by tandemly arranged repeats as HT (head-tail). Head and tail refer to the REP body and the terminal GTAG motif, respectively (see also ref. [9]). Some elements are grouped, and groups may include singletons as dimers arranged in different configurations. The smallest groups are represented by trimers, which can be viewed as singletons next to dimers of different types. Large REP clusters have a variable composition. Most include singletons or dimers reiterated in tandem, along with segments of flanking DNA of variable length. The number of singletons, dimers and grouped elements, vary extensively among GTAG families (Figure 1). Single elements predominate in families 14, 16 and 24 respectively found in *D. alkenivorans*, *Cyanothece* sp. 7424 and *P. stutzeri*. In contrast GTAG-1 families in *P. syringae*, *X. campestris* and *Thauera* sp. Mz1T, the GTAG-3 family in *C. sakazaki*, and all GTAG-23 families are largely made by clustered elements. HH is the privileged type of dimer in most families, but TT dimers outnumber HH dimers in families 1, 3, 19 and 24. HT dimers are absent, or under-represented, in most genomes.

*T. roseum* features two chromosomes, and GTAG-23 elements are distributed in both (Additional file 1).

### CGTC families

**Figure 2:**
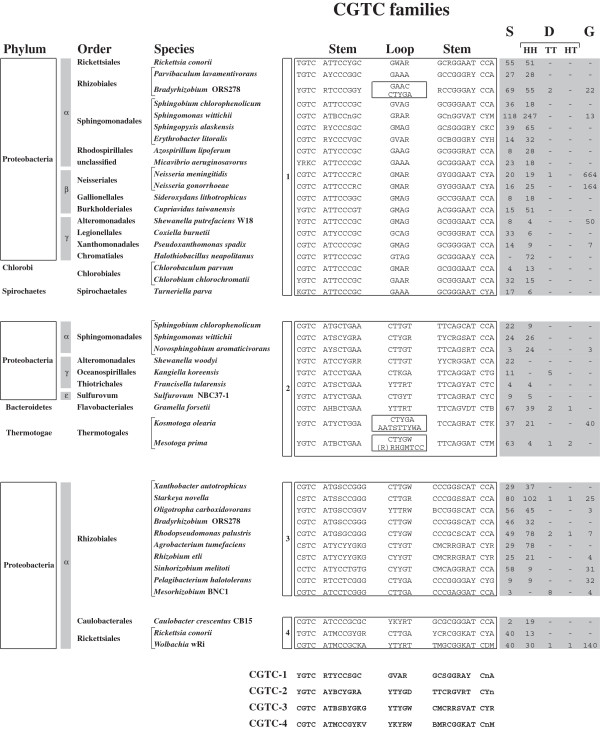
**Families of CGTC repeats.** The consensus sequences of CGTC-1 to CGTC-4 repeat families are reported. Data are presented as in Figure 1. Differences among the four repeat types are highlighted by the all families alignment at the bottom.

CGTC elements are more similar to each other than GTAG elements, and have been assigned to only four families (Figure 2). Differences in sequence and overall structure of the main sequence types are ready to perceive by looking at the all families alignment at the bottom of Figure 2. The terminal CGTC motif is changed to TGTC or CCTC in many repeats. Stems measure 8 (families 1 and 2) or 9 bp (families 3 and 4), and almost invariably feature complementary AT residues in first and second position. Loops measure 4 (family 1) or 5 bp (families 2 to 4), and most fit a few major sequence types. Loops of different length and composition are found in *Bradyrizobium* CGTC-1, and *K. olearia* and *M. prima* CGTC-3 REPs. All CGTC elements end, similarly to GTAG-1 and GTAG-2 repeats, with short unpaired “tails”, most of which fit the consensus CCA.

CGTC repeats have been found in microorganisms belonging to 5 phyla. Most reside in alpha-Proteobacteria, and CGTC REP families have been found in species of all the orders in which the alpha subdivision diverged [32]. The obligate bacterial predator *Micavibrio aeruginosavorus,* which hosts a family of CGTC-1 repeats, has been placed by phylogenetic analyses as a deep branch lineage within the alpha-Proteobacteria, and forms a sister clade to the *Rhodospirillales* order, that is otherwise distinct from the major alpha-Proteobacterial groups currently recognized [33]. Different CGTC REP families coexist in *S. chlorophenolicum*, *S. wittichii*, *Bradyrhizobium* and *R. conorii* (Figure 2).

Five of the species listed in Figure 2 (*S. chlorophenolicum, A. tumefaciens, A. lipoferum, C. taiwanensis* and *S. meliloti*) have either two chromosomes, or one chromosome and one or more megaplasmids. The total number of repeat types in each organism is reported in Figure 2. The number of repeats in chromosomes and megaplasmids is reported in Additional file 1.

CGTC repeats are as heterogeneous as GTAG repeats, as illustrated by the extensive use of the IUB code in Figure 2, needed because several families include subsets made by units having different stem, loop or tail sequences. CGTC elements are predominantly organized as HH dimers. TT dimers are rare, HT dimers negligible. Grouped elements are also rare, but it is worth noting that most of the elements found in *Neisseriae* and *Wolbachia* are organized in large clusters.

Some repeats correspond to described sequences. CGTC-1 elements in *Neisseriae* correspond to the dRS3 repeats [34], CGTC-1 and CGTC-4 elements in *R. conorii* to RPE-6 and RPE-4 repeats [13], respectively. In contrast, the CGTC-4 elements identified in the genomes of the *Wollbachia* endosymbionts of *D. simulans* and *D. melanogaster* are unrelated to the palindromic WPE repeats identified in the *Wolbachia* endosymbiont of *Brugia malayi*[35].

### Association of GTAG and CGTC to other repeats

The diversity of flanking DNA suggests that most REPs are not associated to other sequence repeats. We have not investigated this issue in detail, because out of the scope of this paper. Yet, it is worth mentioning that members of a few REP families repeats are regularly associated to similar DNA tracts. Many *A. variabilis* GTAG-15 dimers are inserted within long palindromic sequences fitting the consensus TATAGGAnTnnnATTTGATTnnTGAAA••TTTCAnnAATCAAATnnnAnTCCTATA (capital letters denote complementary bases, dots GTAG-15 dimers). *T. roseum* GTAG-23 elements are inserted within small palindromes fitting the consensus CCGSSCC (n3, 4) GGSSCGG, all the *H. neapolitanus* CGTC-1 dimers within 41 bp palindromic sequences, fitting the consensus GGGaaGCTT-GAAAaACC••attcacgGGTaTTTCgAAGC-gCCC (letters and dots are as above). Target palindromes unlinked to REP sequences were not found in *A. variabilis* as in *H. neapolitanus* DNA. In contrast, hundreds copies of the GTAG-23 target occur in the GC-rich *T. roseum* genome. Many of the *Neisseria* CGTC-1 elements clustered in large mosaic intergenic regions are interleaved with members of different repeat families [36].

### Variations of GTAG and CGTC families

**Figure 3:**
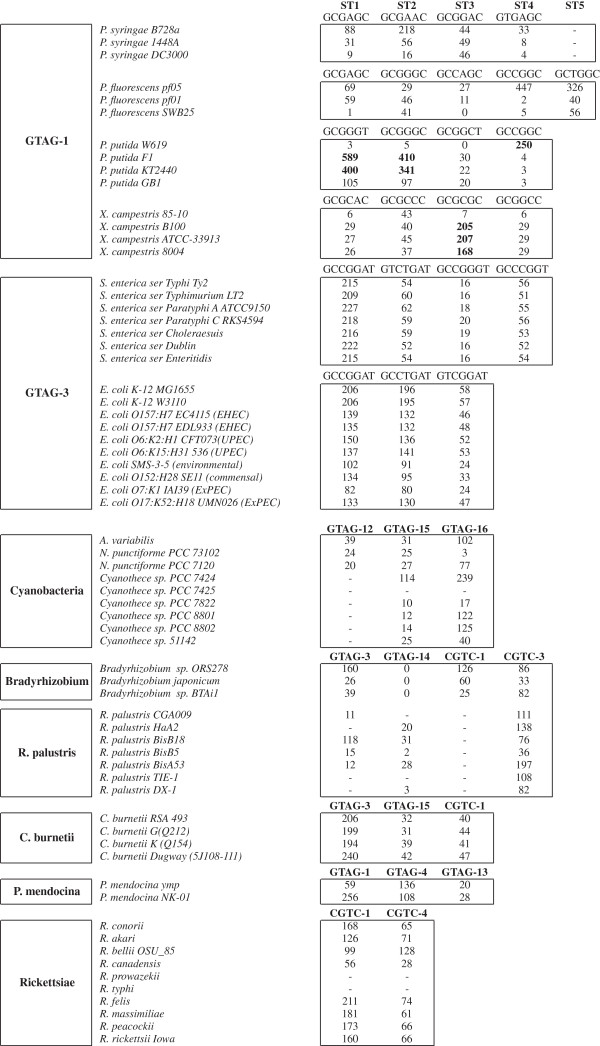
**Strain variations of REP families.** For GTAG-1 and GTAG-3 families, the relative abundance of major sequence types (ST) in the indicated strains are shown. For clarity, of each ST only left-hand, stem sequences are reported. Abundant sequence-subfamilies are highlighted.

The organization of abundant REP families was analyzed in genomes of the same or related species. We monitored the relative abundance of the predominant sequence types (STs), as changes in the distribution of singletons, dimers and grouped elements. Data on species containing one or more REP families are reported in Figure 3. No significative variations were found in families of repeats residing in *P. aeruginosa*, *H. infuenzae*, *S. maltophilia*, *N. meningitidis*, *N. gonhorroeae, C. burnetii*.

Changes in the organization of specific families among strains and/or species are discussed below.

#### Pseudomonas REPs

The compared strains of *P. syringae*[37]*P. fluorescens*[38] and *P. putida*[39] represent major phylogenetic clades, adapted to specific lifestyles and environmental niches. The number of GTAG-1 repeats varied in the genomes examined over a 5–10 fold range, mostly for the expansion of specific repeat sub-populations. The *P. putida* F1 and KT2440 strains are overrun by ST1 and ST2 units, but have few ST4 units, which in contrast are predominant in the W619 strain (Figure 3). Similarly, the large sizes of the GTAG-1 families in *P. fluorescence* Pf-05 and *P. syringae* B728A genomes are correlated to the expansion of ST2 and ST4 units, respectively. Many of these repeats are reiterated in tandem, suggesting that amplification and clustering of REPs may be correlated processes.

#### Enterobacterial REPs

The number of GTAG-3 repeats was comparable in all the strains of *Salmonella enterica* analyzed, but varied over a twofold range among pathogenic, laboratory and environmental *E. coli* strains. The organization of GTAG-3 repeats found in the known MG1655 *E. coli* strain is largely conserved in all the strains analyzed, and size changes of the various repeat families are not correlated to the expansion of specific STs, but rather to an increased number of dimers and clustered elements in MG1655 DNA.

#### Bradyrhizobia REPs

The organization of REP families was monitored in three strains of the genus *Bradyrhizobium*, and six strains of *R. palustris*. *Bradyrhizobium* sp. ORS278 and BTAi1 are photosynthetic bacteria, isolated from stem nodules of different *Aeschynomene* species, *B. japonicum* USDA110 is a non-photosynthetic rhizobium able to form root nodules on soybeans [40]. The relative abundance of GTAG-3, GTAG-14, CGTC-1 and CGTC-3 elements varied over a 8-fold range among the three strains, each repeat peaking in one or two strains only (Figure 3). While comparable in size, GTAG-14 families in *Bradyrhizobium* sp. ORS278 and *B. japonicum* USDA110 significantly differ in their organization. Units with large GCGG/CCGC type loops (see Figure 1) are very few in *B. japonicum* DNA, but the number of HH dimers found in this species is much higher than in *Bradyrhizobium* sp. ORS278 (59 vs 38 dimers).

The size and the pattern of distribution of GTAG-3, GTAG-14, and CGTC-3 families in the six *R. palustris* strains analyzed does not match the hierarchical clustering resulting from the analysis of Pfam domains, according to which BisA53 and BisB18 strains cluster together, BisB5, HaA2, CGA009, and TIE-1 strains on a distinct branch, with CGA009 and TIE-1 on the same node [41]. GTAG-3 elements peak in BisB18, are 10-fold less abundant in other strains, and missing in TIE-1. CGTC-3 elements reside in all strains, but their abundance varied over a 5-fold range, moderately abundant families of GTAG-14 repeats in BisB18, BisA53 and HaA2 strains only.

#### Cyanobacterial REPs

GTAG-15 and GTAG-16 elements were monitored in three filamentous (*Anabaena variabilis*, *Anabaena* sp. strain PCC 7120, *Nostoc punctiforme* PCC 73102) and six unicellular cyanobacteria of the genus *Cyanothece* (51142, 7424, 7425, 7822, 8801 and 8802 strains) showing high genetic variation [42]. Both GTAG-15 and GTAG-16 elements peak in the 7424 strain, are 2–10 fold less abundant in other strains, and are missing in the 7425 strain. Curiously, the DNA of this strain has a GC content significantly higher than the DNAs of the other strains analyzed (49% vs. 37-39%; see ref. [42]). GTAG-12 repeats were detected in filamentous Cyanobacteria only, and are two times more abundant in *A. variabilis* than in *Anabaena* sp. strain PCC 7120 and *Nostoc punctiforme* PCC 73102.

#### Rickettsial REPs

CGTC-1 and CGTC-4 repeat families varied in size over a two-fold range in many species of the genus *Rickettsia*. The lowest number of repeats was found in *R. canadensis*. Neither CGTC-1 nor CGTC-4 elements were found in *R. prowazeki* and *R. typhi*, a result in line with literature data indicating that both species lack repetitive sequences [43].

### Organization of REP dimers

GTAG as CGTC elements are frequently associated to form dimers. The relative abundance of REP dimers im most families is underestimated, as a consequence of both sequence variation and the insertion of DNA between dimer partners. In *P. fluorescence*, most GTAG-1 singletons are remnants of HH dimers [26], and this may hold true for more species upon closer inspection. The components of HH or TT dimers may fold separately, or form a single, large SLS [9,44]. Both HH and TT dimers can be further distinguished because made up by the same elements (homodimers), or elements which feature different stem and/or loop sequences (heterodimers). Further variation was observed in *S. maltophilia*, about 10% of dimers found in this microorganism being heterodimers formed by members of different GTAG families (hybrid dimers; the components of these dimers have been counted as singletons in Figure 1). The number of homodimers and heterodimers varies significantly among REP families. Most HH and TT GTAG-1 dimers in *P. entomophila* and *P. putida* are homodimers. In contrast, GTAG-3 dimers in *Enterobacteriaceae* are exclusively formed by elements with loops of different lengths, and *P. aeruginosa* GTAG-24 dimers by elements with different stems (see changes at stem residues 12 and 13 in Figure 1). Homodimers predominate among CGTC-1, heterodimers among CGTC-2 and CGTC-3 elements. Yet only heterodimers are formed by *H. neapolitanus* and *C. taiwanensis* CGTC-1 repeats, as only homodimers by *N. aromatocivorans* CGTC-2 and *A. tumefaciens* CGTC-3 repeats.

The preferential formation of heterodimers over homodimers in most CGTC and GTAG families has no obvious explanation. Dimers may form large DNA hairpins in single-stranded state or DNA cruciforms. These structures cause replication stalling, and in turn lead to genome instability, and need to be eliminated by specific enzymes during DNA replication [45]. The deletion frequency is significantly influenced by the stability of base pairing involving the first 16–20 bp stem residues [46]. In *E. coli* secondary structures formed by IRs are removed by enzymes of the SbcCD complex, and the minimum duplex stem length necessary for cleavage lies between 8 and 16 bp [47]. These considerations suggest that heterodimers may be protected from enzymatic degradation and genome clearance. Large secondary structures formed by pairing of adjacent REPs may have functional relevance at the RNA level, and differences in the extent of base pairing between homodimers and heterodimers may determine whether the RNA hairpins formed are sensitive or resistant to cleavage by specific endoribonucleases [17,19].

**Figure 4:**
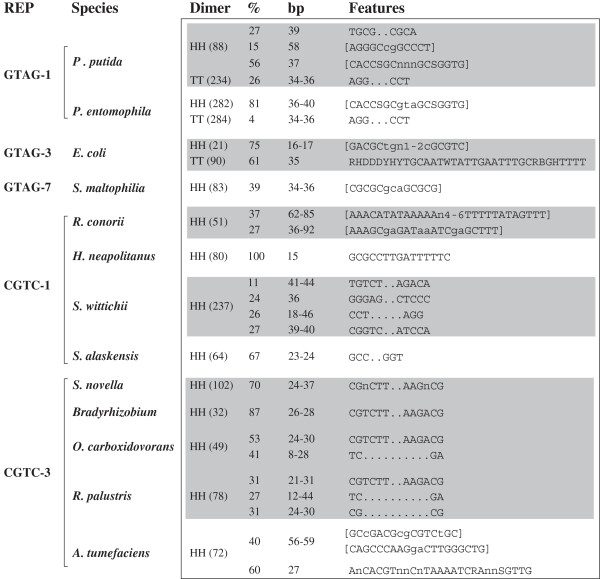
**Spacers in REP dimers.** The organization of spacer sequences in abundant families of dimers is shown. The number of HH or TT dimers [in parentheses] and the relative abundance of the spacer variants are shown. Spacer features include complementary ends or SLSs (in brackets; complementary bases are in capital letters). The two SLSs in *A. tumefaciens* spacers are separated by 20–23 bp. The sequence of the *E. coli* TT dimer spacers is from reference [48].

The distance between dimer partners is variable. Only 1–2 bp separate the partners of *O. terrae* GTAG-17 HH and GTAG-19 TT dimers. The same holds for *Wolbachia* CGTC-4 dimers, and in some both spacer and a few adjacent REP bases have been deleted. In most dimers, spacers vary in length from 20 to 100 bp. Some are largely conserved, others differ in sequence but have similar lengths, or differ both in sequence and size. As a rule of thumb, TT and HH dimers feature variable and conserved spacers, respectively. However, as illustrated in Figure 4, different spacer types may coexist in large dimer families. Several dimers carry spacers which feature either complementary ends, or small SLSs at one end. Two distinct SLSs are at the ends of the spacer in several *A. tumefaciens* CGTC-3 dimers (Figure 4). The presence of structured spacers immediately suggest that dimers may fold into stable hairpins.

It may be of interest noting how the relative abundance of different spacer types may vary among related species. *P. putida* GTAG-1 HH dimers have three types of spacers. Of these, only one is conserved in *P. entomophila* elements, and at lower abundance. The number of GTAG-1 TT dimers in the two species is comparable, but the relative amount of spacers with complementary ends is significantly different.

### Genome distribution of REP sequences

Members of most of the REP families identified are spread throughout the genome. A noticeable exception is represented by *T. roseum* GTAG-23 elements, which are clustered in large blocks at few loci.

**Figure 5:**
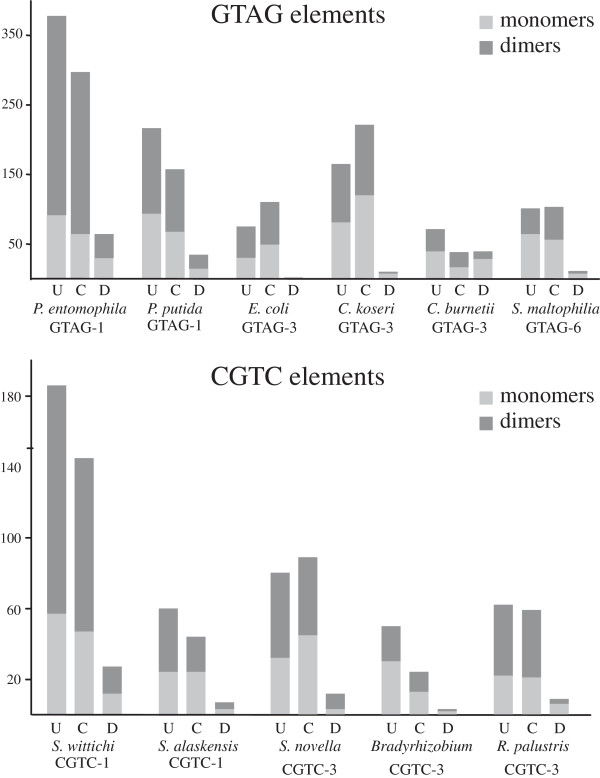
**REPs and flanking ORFs.** The number of single REPs and dimers located between convergently (conv-REPs; C), divergently (div-REPs; D), and unidirectionally (uni-REP; U) transcribed ORFs in different species is shown.

**Figure 6:**
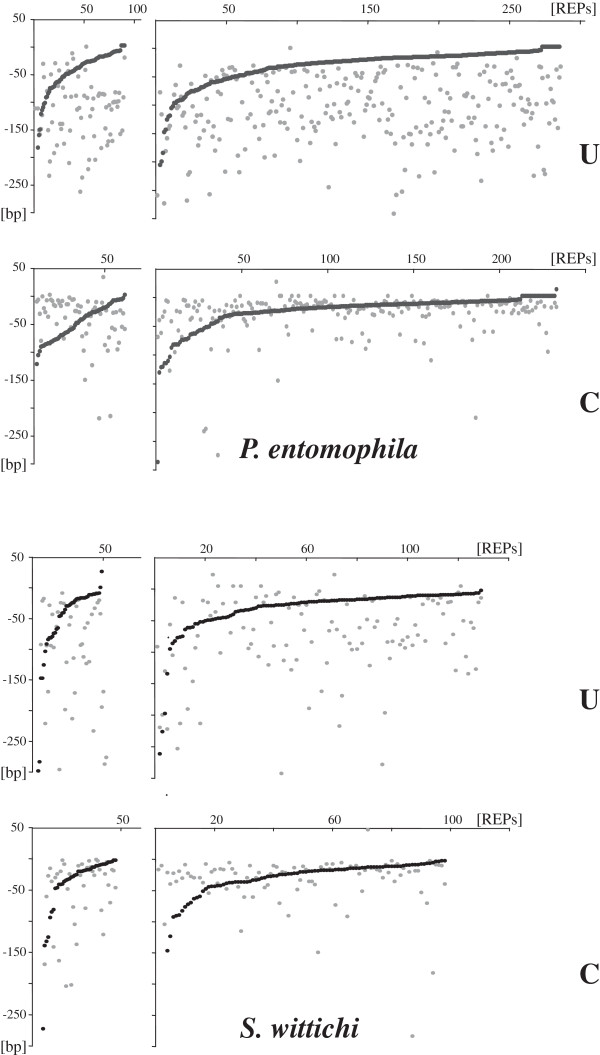
**Distances between REPs and flanking ORFs.** Dots denote the relative distances from flanking ORFs of uni- and conv-REPs of the *P. entomophila* GTAG-1 and *S. wittichi* CGTC-1 families. In the uni-REP graphs, upstream and downstream located ORFs are marked as black and gray, respectively. In the conv-REP graphs, the two upstream ORFs are arbitrarily distinguished by the two color code. Single elements and dimers have been separately analyzed. Distances have been sorted by length to facilitate data visualization.

Most REPs are located in the intergenic space. Relative to the orientation of flanking ORFs, repeats may be located between either convergently (conv-REPs), or divergently (div-REPs), or unidirectionally (uni-REP) transcribed ORFs. In different REP-rich genomes the repeats are predominantly located between unidirectionally and convergently transcribed ORFs (Figure 5). This finding reinforces the notion that most REPs are transcribed, and may function as RNA sequences. The distances separating *P. entomophila* GTAG-1 and *S. wittichi* CGTC-1 elements from flanking ORFs are diagrammed in Figure 6. The pattern of interspersion of singletons and dimers, separately analyzed, is similar. In *P. entomophila* as in *S. wittichi*, most conv-REPs are next (<20 bp) to the 3′ end of both flanking ORFs. Uni-REPs are also located close to the 3′ end of upstream ORFs, but are at varying distances from downstream ORFs. This suggests that the fraction of readthrough transcripts spanning REPs, that may influence the expression of both flanking ORFs, may be limited. The pattern of interspersion of GTAG-1 and CGTC-1 elements and flanking ORFs did not vary in other REP-rich genomes analyzed (Additional file 2).

**Figure 7:**
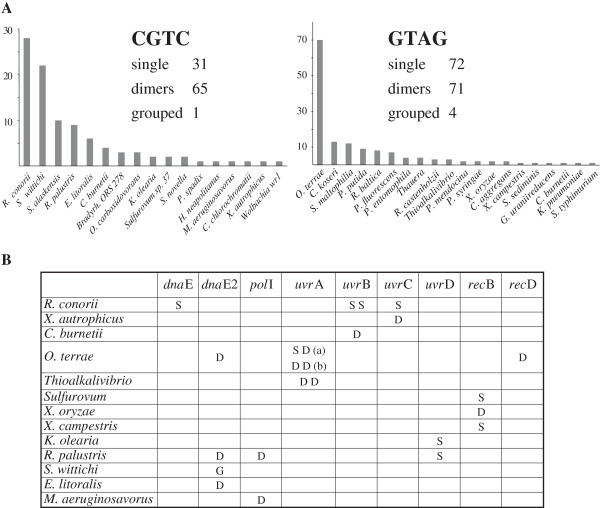
**Intragenic REPs. A)** ORFs interrupted by CGTC and GTAG elements in different species **B)** DNA synthesis and repair genes carrying REPs. S, G and D denote single, grouped elements and dimers, respectively. The *R. conorii uvr*B and the *Thioalkalivibrio* uvrA genes are interrupted at different sites by two single REPs and two REP dimers, respectively. The two *uvr*A genes found in *O. terrae* are both interrupted by double REP insertions, either a single REP and a REP dimer (a, ORF 2709), or two REP dimers (b, ORF 3168).

Members of several REP families are close to, or even overlap coding regions. The extent of contiguity is immediately illustrated by the finding that the termini of GTAG REPs often provide the opal stop codon (TAG) to flanking ORFs. In different species, a variable number of REPs are entirely located within ORFs. Target ORFs and REP-encoded amino acids are listed in Additional file 3, data are summarized in Figure 7. In all the genomes examined, a plethora of regions, selected on the base of arbitrary length thresholds, have been annotated as ORFs, but encode short proteins plausibly all spurious. Therefore, REPs mapping within hypothetical proteins <120 amino acids have been not included in the pool of intragenic elements.

The highest number of intragenic GTAG and CGTC repeats were found in *O. terrae* and *R. conorii*, respectively (Figure 7A). Intragenic *R. conorii* repeats correspond to the described RPE-4 and RPE-6 elements [13], and is worth recalling that other genes are interrupted in this species by longer palindromic insertions called RPE-1 [14]. More than 50% of the inserts are dimers or grouped repeats, which encode 20 to 30 amino acids. In some *O. terrae* and *R. conorii* ORFs, single elements and/or dimers are inserted twice, at close or distant sites. Larger REP-encoded regions have been found in *Thauera* and *R. conorii*, where clusters of repeats encode 43 to 82 amino acids (Additional file 3). The remaining elements are variably located along ORFs. Slightly more than 10% of GTAG and CGTC repeats are at the end of the coding region, a higher number at the ORF NH2 terminus. Of these, many may be extragenic, since translation may initiate not at the predicted, but rather at downstream sites. As inferred by alignment to shorter homologous proteins encoded by either related species, or strains of the same species, most REPs located within the 5′ end of *P. putida*, *C. koseri* and *S. maltophilia* ORFs may be not codogenic, but rather function as post-transcriptional control elements. On the other hand, *R. conorii* proteins decorated by RPE-1 elements at the NH2 terminus are expressed in vivo [49]. Would we ignore all ORFs carrying REPs in the NH2 terminus, the number of ORFs decorated by REPs is still high.

The encoded proteins belong to different categories, but many play a role in DNA synthesis and repair. Different species potentially encode REP-decorated proteins involved in nucleotide excision (excinuclease ABC complex proteins, UvrD/REP helicase, DNA polymerase I), or in homologous recombination repair (*rec*BCD proteins; Figure 7B). The two *uvr*A genes found in *O. terrae* are both interrupted at different sites by dual REP inserts. REP-tagged proteins include the inducible, error prone DNA polymerases, encoded by DnaE2 genes [50]. In *R. conorii*, which lacks DnaE2, a REP element is inserted within the DnaE gene, which encodes the high-fidelity replicative polymerase (Figure 7B). Remarkably, some of the listed ORFs are the only coding sequences modified by REPs in a given species. REPs are also inserted in other genes involved in DNA repair, such DNA ligase in *O. terrae*, a DNA-photoreactivating enzyme in *Thauera*, as in genes encoding RNA binding proteins, such RNA helicases in *O. terrae*, tRNA synthetases in *X. oryzae, E. lithoralis* and *S. alaskensis*, tRNA pseudouridine synthase B subunit genes in *S. maltophilia*, *E. lithoralis* and *S. alaskensis*. Curiously in *S. maltophilia*, also the A subunit gene is interrupted by a REP (Additional file 3). In light of these findings, may be worth recall that the *R. conorii* tRNA pseudouridine synthase B subunit gene is interrupted by RPE-1 sequences [14].

Sequence alignment revealed that the different REPs within *X. campestris* and *X. Oryzae* recB genes are located about at the same site in the coding region. In contrast, REPs found in other genes belonging to the same functional category are inserted at different sites.

### REPs and tyrosine transposases

GTAG repeats are often found close to genes encoding tyrosine transposases denominated RAYTs [25]. The genetic elements resulting from the association of RAYT and REP sequences are known as REPtrons [51]. REPtrons have been identified in most of the species hosting GTAG repeats listed in Figure 1, as well as in species lacking GTAG repeats (Additional file 4). REPtrons may be missing in some species, because eliminated by deletion as described for many *E. coli* strains [51].

**Figure 8:**
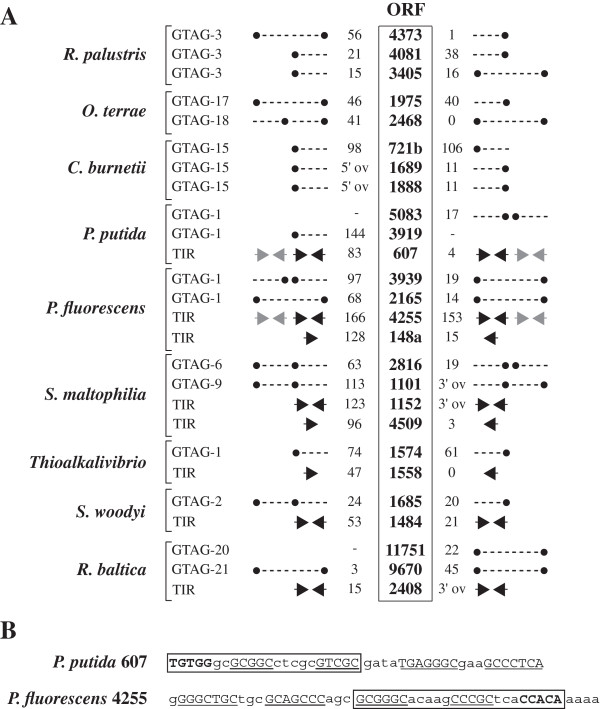
**Tyrosine transposase genes. A)** Different tyrosine transposase genes are flanked by REP sequences, either monomers or dimers (−−-•), or by unrelated inverted repeats (→) at the indicated bp distances. 5′ov and 3′ov refer to flanking sequences overlapping tyrosine transposase genes at the 5′ or 3′ end, respectively. **B)** The sequences of the double inverted repeats flanking *P. putida* 607 and *P. fluorescens* 4255 are reported. Palindromic residues are underlined, degenerated GTAG-1 sequences are boxed.

**Figure 9:**
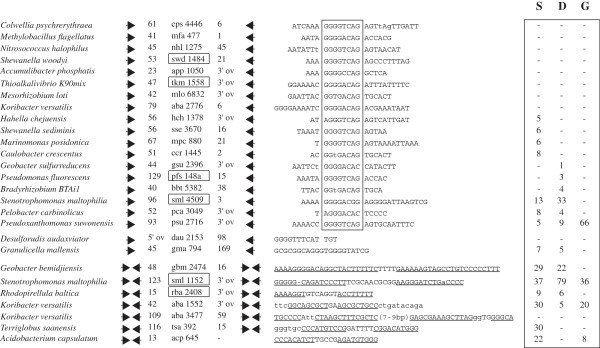
**TIRYT genes.** TIRs flanking TIRYT genes are diagrammed as arrows. Distances in bp separating genes and TIRs are shown, 5′ov and 3′ov refer to overlapping flanking sequences as in Figure 8. Only upstream TIR sequences are shown. Lower case letters denote non complementary TIR residues. ORFs shown in Figure 8, and conserved GGGGWSAS motifs, are boxed. Complementary residues in the double palindromic TIRs shown at the bottom are underlined. Boxed numbers to the right refer to single (S), dimeric (D) or grouped (G) TIR sequences found in the indicated genomes.

Species that have multiple GTAG repeats families feature also repeat-specific REPtrons. It is of interest noting that species hosting only one REP family often feature multiple REPtrons. In these, transposase coding sequences, organization and relative position of flanking REPs all vary (Figure 8A; see also Additional file 4). Curiously, REPs are replaced in some REPtrons by long TIRs. TIRs flanking *P. putida* ppf 607 and *P. fluorescens* pfs 4255 ORFs result from the adjoining of degenerated GTAG-1 units to unrelated SLSs (Figure 8B), and hundreds of these bizarre structures were found in *P. putida* and *P. fluorescens* genomes. In contrast, all other TIRs shown in Figure 8A are unrelated to REPs. RAYT genes identified in species that lack GTAG REPs are similarly flanked by TIRs (Figure 9). All these genetic elements and the encoded transposases have been called in accordance TIRtrons and TIRYT (TIR associated tyrosine transposase), respectively. Some TIRs are located about at the same distance from transposase coding sequences, and are plausibly variants of one or a few sequence types, as they share a motif fitting the consensus GGGGWSAS (Figure 9). Other TIRs are unrelated to each other, and some include partly or wholly self-complementary tracts. Moderately abundant families of TIRs have been identified in some microorganisms. Many TIR elements are organized as REPs in dimers or clusters (Figure 9). The highest number of TIR repeats was found in the *S. maltophilia* K279a strain*,* which hosts two TIR families, corresponding to the two TIRYT genes ORFs 1152 and 4509. The 1152 and 4509 TIR repeats markedly differ because the former are self-complementary, and are predominantly found at short distance from each other. TIR families of comparable size and organization were found in the other wholly sequenced *S. maltophilia* strains R551-3, JV3 and D457. *Koribacter versatilis* has three TIRYT genes (ORFs 1552, 2776, 3477) decorated by different TIRs. Only ORF1552 TIRs are members of a repeated DNA family.

Some of the identified RAYTs, and all the TIRYTs listed in Figure 9, have been aligned for comparison (Additional file 5). The catalytic tyrosine and the HUH (hystidine-hydrophobic-hystidine) domain, typical of transposases of the IS200/IS605 group, are conserved in all, as well as motifs distinguishing RAYTs from bulk IS 200 transposases [25] and other amino acids at several positions. RAYTs and TIRYTs are distinguishable for length and amino acid signatures, and TIRYTs can in turn be assigned to four main groups (Additional file 5). Of these, the more sharply defined is represented by the transposases encoded by *T. saanensis* (tsa 392), *K. versatilis* (aba 1552 and 3447), *A. capsulatum* (acp 645) and *G. mallensis* (gma794), species all belonging to the Acidobacteria phylum.

In spite of the overall similarity to GTAG elements, CGTC repeats are not associated to transposase genes. Many of the CGTC-positive species in Figure 2, among which *Bradyrhizobium sp*. ORS278*, C. crescentus, C. taiwanensis, G. forsetii, R. palustris. Sulfurovum* sp. NBC37-1*, K. olearia, P. spadix, S. lithotrophicus*, encode tyrosine transposases, but none of the corresponding genes were flanked by CGTC sequences. The interspersion of CGTC elements with other classes of transposase genes was also monitored, but only a few fortuitous associations have been detected.

## Discussion

Data reported in this work support the notion that many short palindromic repeats found in prokaryotes may be evolutionarily related, and catalogued as members of two large DNA super-families alternatively tagged at one end by GTAG or CGTC motifs not involved in base pairing. Distinctive features of GTAG and CGTC repeats are summarized in Table 1. GTAG and CGTC super-families include more sequence classes than those reported. Members of either type may have escaped detection because: 1) smaller than average repeats. *Thauera* GTAG-1 elements, which feature only 5 bp stems, were fortuitously discovered by inspection of the tandem repeat database [52] 2) unusual in structure, for the presence of bulges due to unpaired residues 3) poorly recognizable, as the degenerated Pseudomonas GTAG-1 repeats shown in Figure 8. The data presented are however sufficient to draw a coherent picture of the organization of GTAG and CGTC repeats, evaluate the pattern of distribution of the various families among species, reexamine the roles that these sequences may play, shed light on the processes by which they might have been formed.

**Table 1:** Features of REP families

	**GTAG**	**CGTC**
GW extra-bases	GTAG-1, 2	-
3 bp tail	GTAG-1, 2	all
stem length	5-13 bp	8-9 bp
loop length	2-20 bp	4-5 bp
clusters	frequent	rare
HH dimers	predominant	predominant
TT dimers	frequent	rare
intragenic units	+	+
association to TPAse genes	+	-

GTAG and CGTC REP families vary in size over a 50-fold range, some including thousands units, many 20–100 units, or even less, and are unevenly distributed among species. Both observations rule out that these elements may be important chromosome components fulfilling the same general functions in all organisms [8,10]. In contrast, the beneficial effects on host fitness may vary in different environments, and in some microorganisms specific repeats may just be parasitic DNA. GTAG and CGTC elements come in different chromosomal arrangements. The relative abundance of single, paired and clustered elements within each family varies among species, as among isolates of the same species, and changes in the organization of family units are genomic fingerprints exploitable for genotyping assays [53].

Most of the described REPs are located in the intergenic space. Taking into account that the average intergenic space in prokaryotes is _˜_100 bp [54], many are close to, or overlap with coding regions. The preferential location between unidirectionally and convergently transcribed ORFs, and the frequency of GT pairing of stem residues, both support the notion that many repeats are transcribed, and may function as post-transcriptional control sequences, by tuning the levels of expression of flanking genes.

REPs may as well function as DNA elements. The *E. coli* REPs are targeted by the DNA gyrase [10], and cleavage of REPs located at ORF 3′ ends by gyrase may relieve the excess of supercoiling induced by transcription [55]. This regulatory mechanism would however be effective only in REP-rich species. Other repeats may function as promoters in specific microorganisms and/or genomic contexts. The issue has not been tackled, because promoter analyses without experimental support are merely speculative. Yet, it is worth noting that, analyzing the interspersion of GTAG-1 elements with coding regions in the exopolysaccharide (EPS)-producing bacterium *Thauera* sp. MZ1T, we unexpectedly found that clustered genes involved in EPS synthesis and transport [56] are immediately flanked by arrays of GTAG-1 repeats, which likely direct or modulate their expression.

In different organisms GTAG and CGTC REPs have been found within coding regions, most of which encode known proteins. It is difficult to assess whether intragenic elements may affect the activity of the decorated proteins. The insertion of REPs in a variety of unrelated proteins argues against functional constraints, and genes inactivated by REP insertions have been plausibly removed from the population. Amino acids encoded by intragenic elements found at the NH2- or the COOH-terminus may not affect the function of the protein. Moreover, most REPs located in the NH2-terminal coding region may be extragenic, because of genome misannotation. An additional argument against the inactivating role that REP insertions may play is that tagged proteins may have modular structure, and insertions may be neutral in effect, because located in flexible linkers or loops. In spite of all these cautions, it is difficult hypothesize that genes encoding different proteins involved in replication and global genome repair (UvrABCD and recBCD proteins, DNA polymerase I, error prone DNA polymerases) may have been just fortuitously targeted by REP insertions, also because they are, in many species, the only examples of REP-tagged coding sequences. It is therefore tempting to speculate that insertions may have modified the activity of the mentioned proteins, contributing to the development of hypermutable or mutator microorganisms, which may experience increased recombination, mutation, gene loss, horizontal gene transfer. Multiple tRNA pseudouridine synthase genes also carry REP sequences, but is unclear how these insertions may affect cell physiology. Pseudouridine synthases are involved in posttranscriptional modifications of cellular RNA, but act also as RNA chaperones, a function which may be more important than pseudouridylation per se [57].

The occurrence in multiple distant phyla supports the notion that both GTAG and CGTC repeats are ancient components of the bacterial genome. Most elements reside in Proteobacteria, and GTAG and CGTC repeats have been predominantly identified in the gamma and alpha division, respectively. However, families of either repeat type have been identified in deeper branching phyla among which Termotogae and Planctomycetes, plausibly the deepest branching phylum within the bacterial domain [58]. Planctomycetes cluster with Verrucomicrobia in the PVC superphylum, and *O. terrae*, which belongs to Verrucomicrobia, is highly enriched in GTAG repeats. Bacterial phyla are related to each other linearly, and major evolutionary changes within Bacteria have taken place in a directional manner [28]. REPs plausibly appeared early in evolution, and have been massively lost in time, and maintained in a limited number of microorganisms. How all this occurred is a matter of speculation. Though the actual scenario will likely be modified by analyzing a wider set of genomes, the distribution of REPs described in this work among phyla, orders, families and species is manifestly uneven. GTAG repeats have been identified in microorganisms belonging to 10 of the 15 orders of gamma-Proteobacteria (Figure 1). In turn, only one of a few species within each order host GTAG repeats. Enterobacteria have been subdivided into three clusters on the basis of the character states of aromatic amino acid biosynthesis [59]. Cluster 1 includes *Escherichia*, *Shigella*, *Citrobacter*, *Salmonella*, *Klebsiella*, *Enterobacter*, cluster 2 *Serratia* and *Erwinia*, cluster 3 *Edwardsiella*, *Yersinia*, *Proteus* and *Providencia*. GTAG-3 families are sharply confined to species of enterocluster 1. Similarly, GTAG repeats reside only in some species of the genus *Shewanella*. *Shewanellae* fall into two major clusters based on their 16S rDNA sequences as well as phenotypic properties [60]. Cluster I includes cold-adapted obligate marine species retrieved from the deep sea, cluster II non-obligate marine species retrieved from different environments. Interestingly, GTAG-1 and GTAG-2 families have been identified only in species (*S. sediminis, S. halifaxensis*, *S. pealeana*, *S. woodyi* and S. *piezotolerans*) belonging to cluster I. The above reported examples suggest that the presence/absence of specific REP families may represent a resource exploitable to catalogue bacteria, useful to support, or weaken, phylogenetic relatedness among groups of microorganisms inferred by the use of conventional parameters. CGTC repeats are unevenly distributed among species as well. As an example, CGTC repeats have been identified in all orders of the alpha subdivision, but are missing in several alpha-Proteobacteria, among which bacteria belonging to the families of Acetobacteraceae, Bartonellaceae and Brucellaceae.

The abundant families of GTAG repeats are restricted both in *S. maltophilia*[9] and *P. syringae*[61] to core genome regions. Yet, the spotty distribution is compatible with the hypothesis that specific genomes may have been colonized by REPs as a consequence of HGT (horizontal gene transfer) events. According to this view, repeats must have been acquired along with genes ensuring their multiplication. Differences in the distribution and abundance of REPs among different species, or strains of the same species, are typical of mobile DNA. Different groups in the recent past suggested that REPs are selfish elements propagated by transposition. A key role in the process is (or has been) played by specific tyrosine transposases called RAYTs. Transposon-like elements including REP and RAYT sequences called REPtrons have been identified in a variety of species, regardless the presence of a corresponding REP family. Whether the expression of RAYTs in these elements is driven by REPs is unknown, but marked differences in the organization of REPtrons, as the inability of REPtrons to self-propagate, do not support such hypothesis. The expression of RAYTs is plausibly correlated to the formation of upstream readthrough transcripts, and can be indeed down-regulated by hairpins formed by REPs, which may either promote mRNA degradation, or affect mRNA translation, as observed for IS200 transposases [62]. Direct involvement of RAYTs in the formation of REPs is supported by experiments showing that a recombinant *E. coli* RAYT recognizes single-stranded REP DNA, and cleaves the GTAG motif [51,63]. Cleavage was abolished by mutating the motif, or changing the AA/GC residues at the edges of the loop region (see Figure 1) into paired AA/TT residues, thus by increasing the strength of the REP palindrome. In the model proposed [51] REP sequences are the products of RAYT-mediated excision and recombination events, and HH or TT dimers, or complex REP arrays may result from alternative processing of circular intermediates carrying REP units. GTAG-1 and GTAG-2 repeats carry conserved 3-bp sequences at the untagged end. Whether these “tails” are recognized by RAYTs, and similar signals are present but have been variously altered in other repeat families remains to be established.

Comparative analyses revealed that several RAYT-like genes are not flanked by REPs, but rather by TIRs of different length and composition. These transposases and the corresponding genetic structures have been called for consistency TIRYTs and TIRtrons, respectively. TIRtrons occur in species which contain REPs, but are predominant in species which lack REPs. Given the extraordinary high number of annotated tyrosine transposase genes (at the moment, >2000), it is likely that many REPtron- and TIRtron-like entities occur. Unravelling the complexity of this variegated universe of sequences is out of the scope of this work. Yet, monitoring TIRtrons and similar entities may shed light on the process of formation of REPs, since TIRs flanking some TIRYT genes are members of previously undiscovered repeated DNA families. The formation of TIR and GTAG REP families could thus be mediated by TIRYTs and RAYTs, and occur in an analogous manner. In contrast to REPtrons and REPs, TIRtrons and TIRs coexist in a limited number of genomes, suggesting that TIRYTs may be less productive players than RAYTs.

There is no obvious correlation between the presence of tyrosine transposase genes and the occurrence of REP or TIR families. *K. versatilis* has three distinct TIRYT genes (ORFs aba 2776, 3477, and 1552; see Figure 9), and one family of TIR repeats, *A. phosphatis* two different TIRYTs, ORFs app 1050 (Figure 9) and app 3234 (not shown), but no TIR repeats. In contrast, a plethora of tyrosine tranposase genes and corresponding flanking repeats was found in *P. fluorescens*, *R. baltica* and *S. maltophilia*. This suggests that the formation and/or maintenance of repeats promoted by tyrosine tranposase may be favored in specific microorganisms.

Functional interactions of recombinant RAYTs and TIRYTs with REP and TIR targets may be eventually analyzed to check whether RAYTs can bind and/or cleave TIR repeats, and vice versa, whether TIRYTs recognize GTAG repeats. The variety of REP and TIR targets, and the occurrence of a multitude of element-specific transposases, make *S. maltophilia* a reference organism to set up in vitro assays. For the same reasons, it should be of interest to assess the mobility of GTAG and TIR repeats by population sequencing, as elegantly done to monitor transposition of GTAG-1 repeats in Pseudomonas [26].

CGTC elements markedly differ from GTAG repeats because seem lacking a dedicated transposase. Genes encoding RAYT and other IS200 transposases reside in many of the species carrying CGTC repeats, but none of them is flanked by CGTC units. Such marked difference between GTAG and CGTC elements could be explained by hypothesizing that CGTC REPtrons may have early disappeared, plausibly because able to propagate very efficiently, and therefore highly deleterious to the host. According to this view, the formation of novel repeats is blocked, and CGTC families are going toward extinction. Alternatively, the absence of a dedicated enzyme may imply that CGTC elements can be mobilized by a broad spectrum of transposases. The two hypotheses are not in contrast, and CGTC-specific transposases may have been replaced by functionally related enzymes.

## Conclusions

The provisional framework provided by this paper sets the base for a coherent classification scheme according to which catalogue several small palindromic repeats found in prokaryotes. Future work should clarify the degree of relatedness of CGTC and GTAG repeats, assess whether they have been formed by similar processes, and if such processes are still operative. The relatedness of tagged and untagged SLSs also needs to be investigated. Families of REP-like sequences lacking conserved terminal motifs have been identified in *M. tuberculosis* and *D. radiodurans*[8]*, Bordetellae*[64], *Brucellae*[44] and Cyanobacteria [65], but many more likely occur. It will be of interest to assess whether classes of untagged palindromic repeats may be evolutionarily related, and functionally associated with specific DNA- or RNA-binding proteins.

## Methods

### DNA analyses

DNA sequences analyzed in this work include known and novel repeats. The names and the NCBI accession numbers of all the genomes analyzed in this study are listed in Additional file 6. Novel repeats have been identified by BLAST, using as queries known REPs variously modified, or sets of 20 mers featuring 7–8 base paired residues, separated by loops of variable lengths. Some repeats were identified by searching abundant, self-complementary sequences in individual prokaryotic genomes by using the TRDB (Tandem Repeats Database) facility [52].

The organization of the various repeat families was assessed by using the Fuzznuc program of the EMBOSS package. Genomes of interest were searched for SLSs homologous to queries known or derived from BLAST searches, containing mismatches and a variable number of loop residues. In the pruning procedure, palindromic repeats containing more than one mismatch in the paired region were discarded, but retained when repeats were partners of dimers. GT pairing between stem residues was allowed. Repeats with loops unusual for length or composition relatively to the majority of family members were also discarded. The extent of variation of REP families among different species, or isolates of the same species, was determined by comparing the relative abundance of the major sequence types or subsets identified in representative genomes.

## Abbreviations

bp: Base pair; BIME: Bacterial interspersed mosaic element; BLAST: Basic local alignment sequence tool; CRISPR: Clustered regularly interspaced short palindromic repeat; EPS: Exopolysaccharide; HGT: Horizontal gene transfer; HUH: Hystidine-hydrophobic-hystidine; IS: Insertion sequence; Kb: Kilo base; MITE: Miniature inverted-repeat transposable element; ORF: Open reading frame; PVC: Planctomycetes, verrucomicrobia and chlamydiales; RAYT: REP- associated tyrosine transposase; REP: Repetitive extragenic palindrome; RPE: Repetitive palindromic element; SLS: Stem-loop sequence; ST: Sequence type; TIR: Terminal inverted repeat; TIRYT: TIR-associated tyrosine transposase; TRDB: Tandem repeats database.

## Competing interests

The authors declare that they have no competing interests.

## Authors’ contributions

PPDN conceived the study and wrote the manuscript, FR analyzed the composition of REP families, EDG analyzed intragenic elements and dimer repeats, and prepared all graphic work. All authors read and approved the manuscript.

## Supplementary Material

Additional file 6 Full name and NC accession number of the analyzed strains. Click here for file

Additional file 1 **Distribution of specific repeats in genomes carrying multiple chromosomes.** The distribution of members of specific repeat families in genomes carrying either two chromosomes, or a chromosome and one or more megaplasmids is shown. Click here for file

Additional file 2 **Distance between REPs and flanking ORFs in REP-rich species.** Distances separating REPs from flanking ORFs in four REP-rich species (*P. putida*, *C. koseri*, *S. novella* and *S. alaskensis*) are shown. Data are presented as in Figure 6. Click here for file

Additional file 3 **Intragenic REPs.** The number, the size in amino acids and the hypothesized function of ORFs carrying GTAG and CGTC elements are shown. For each, the interval encoded by REP sequences and the corresponding amino acids are shown. Click here for file

Additional file 4 **REPtrons list.** Tyrosine transposase genes not included in Figure 8 are shown. The sequences of REP-like elements decorating REPtrons found in species lacking REP families are also shown. Click here for file

Additional file 5 **Alignment of RAYT and TIRYTs.** Some of the identified RAYTs, and all the TIRYTs listed in Figure 9, have been aligned for comparison. Click here for file
