# Supplementary material for: GTAG- and CGTC-tagged palindromic DNA repeats in prokaryotes
Source: BMC Genomics. 2013 Jul 31;14:522. doi: 10.1186/1471-2164-14-522 (PMC3733652; doi:10.1186/1471-2164-14-522)
Supplement: Additional file 2 — Distance between REPs and flanking ORFs in REP-rich species. Distances separating REPs from flanking ORFs in four REP-rich species (P. putida, C. koseri, S. novella and S. alaskensis) are shown. Data are presented as in Figure 6. [file 1471-2164-14-522-S2.pdf]

Additional file 2. Distance between REPs and flanking ORFs in REP-rich species. Data are presented as in Fig. 6.

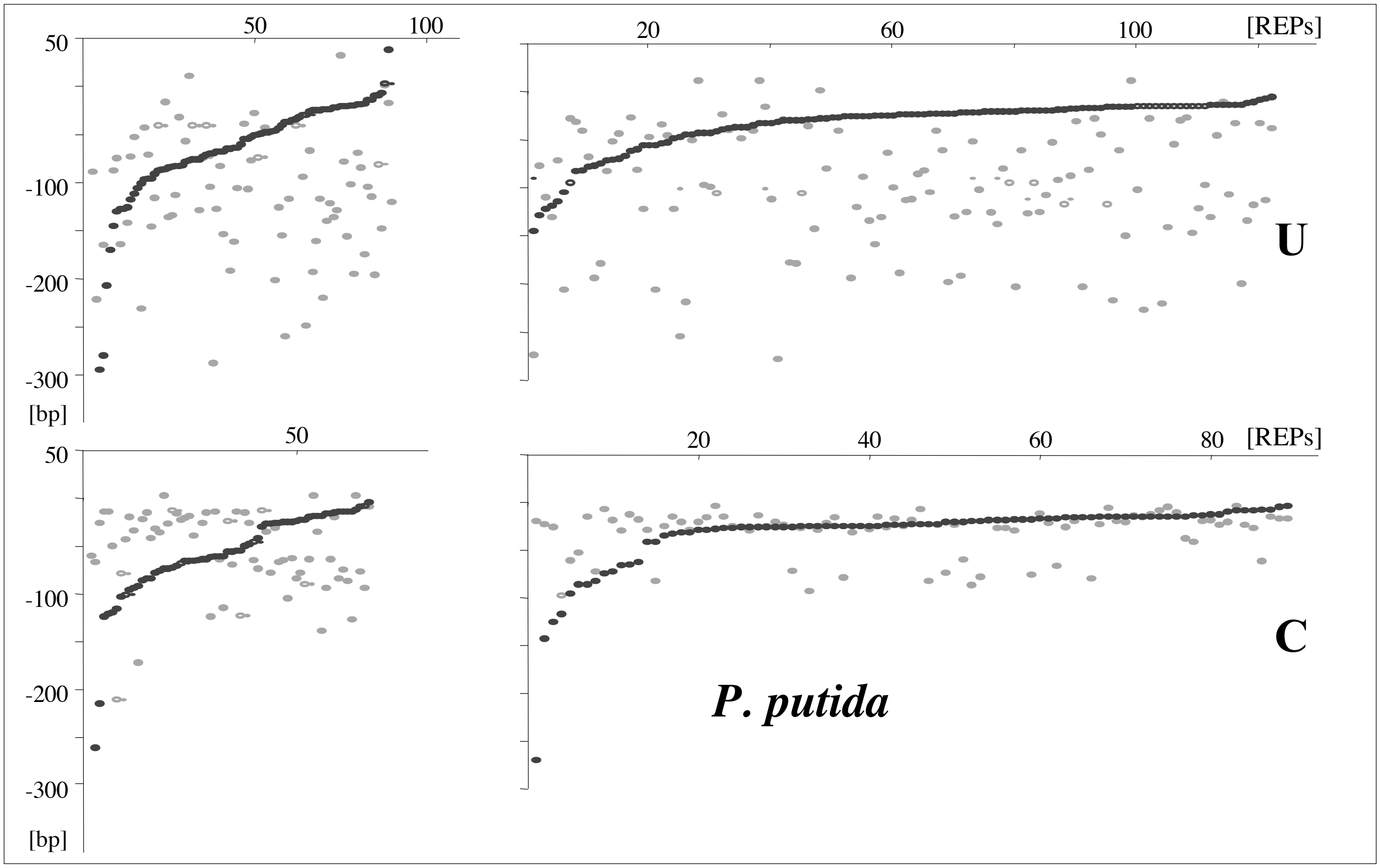

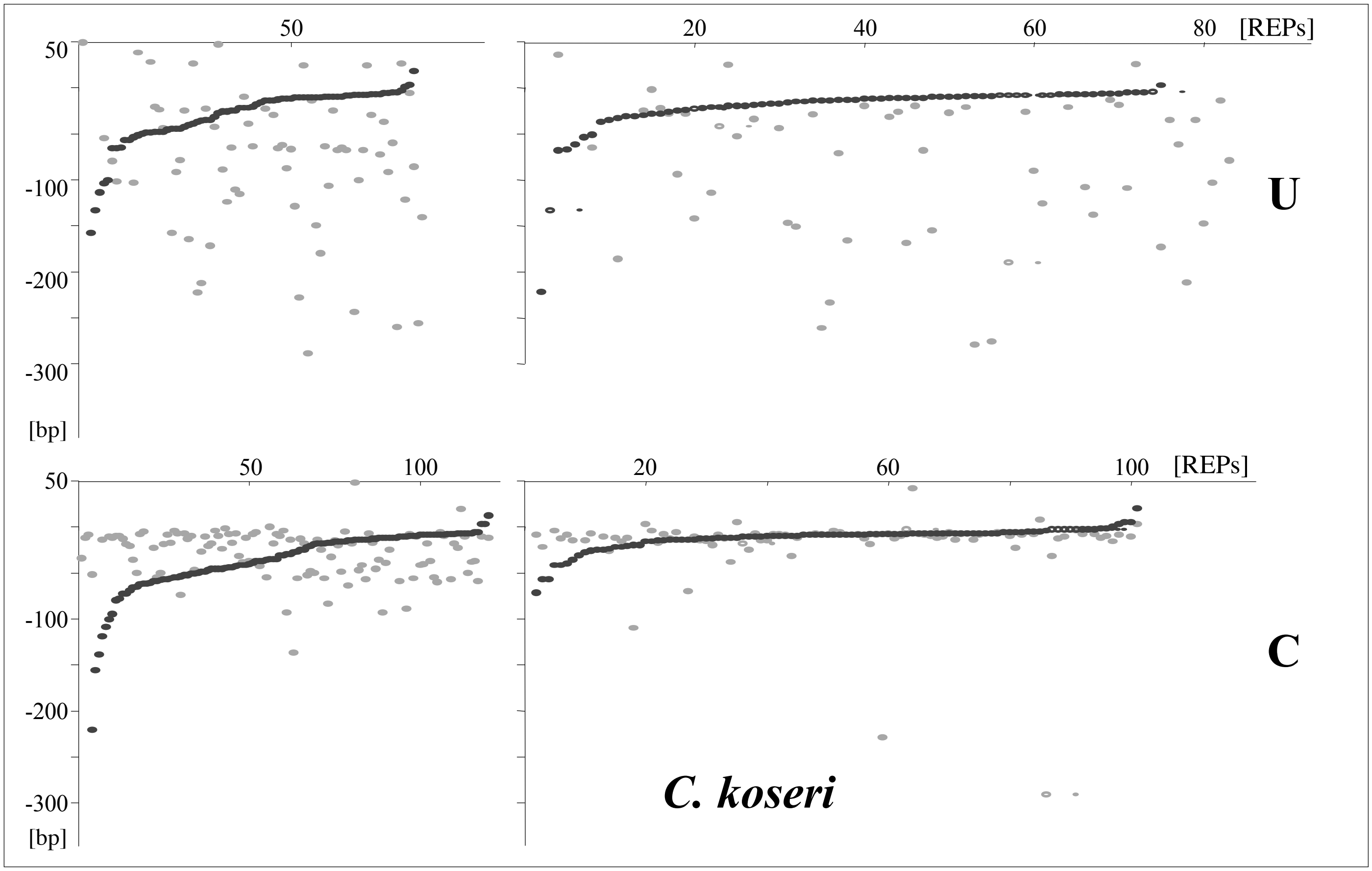

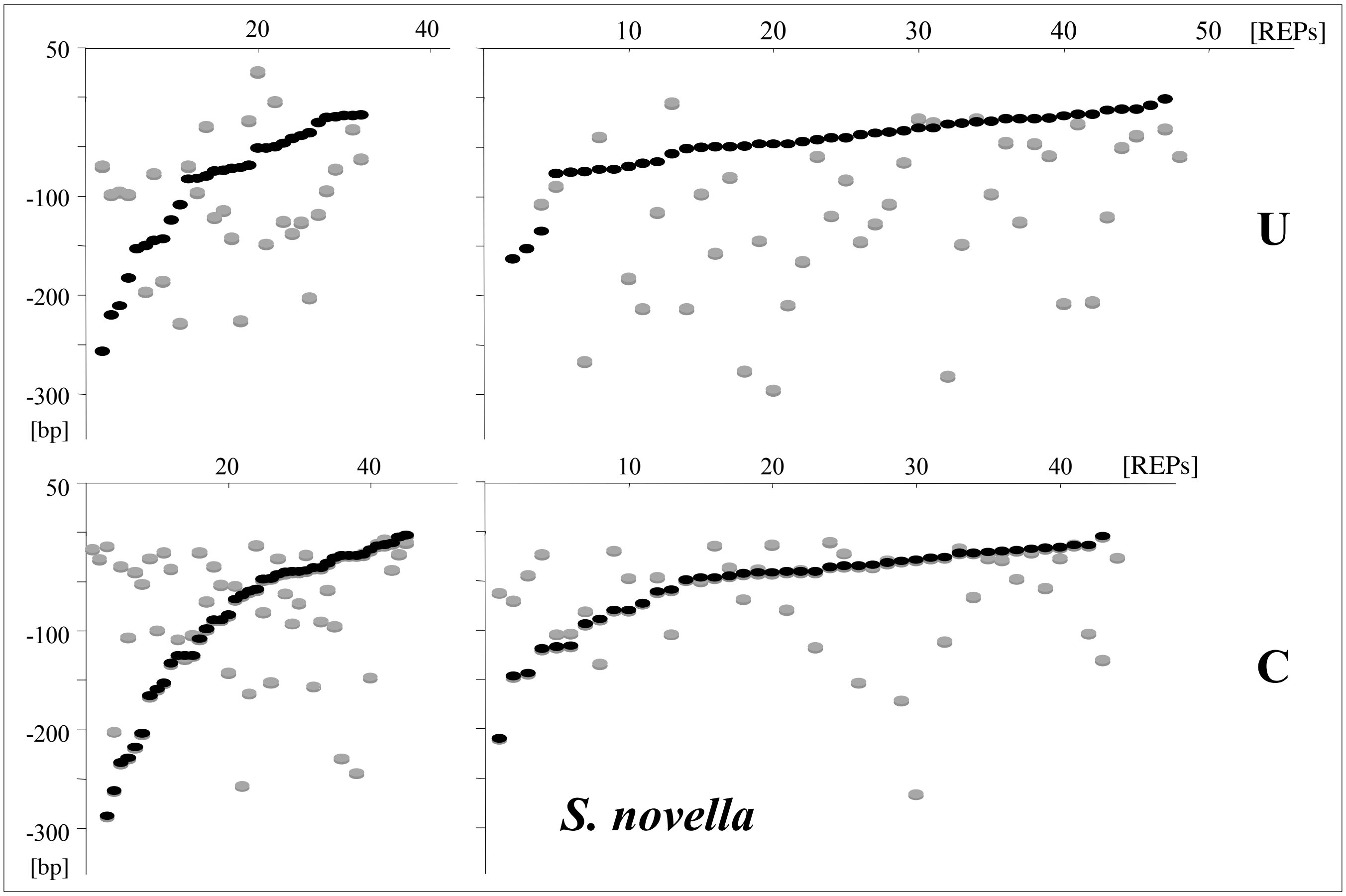
20
40
10
20
30
40
50
[REPs]
50
-100
-200
-300
[bp]
U
20
40
10
20
30
40
[REPs]
50
-100
-200
-300
[bp]
C
S. novella

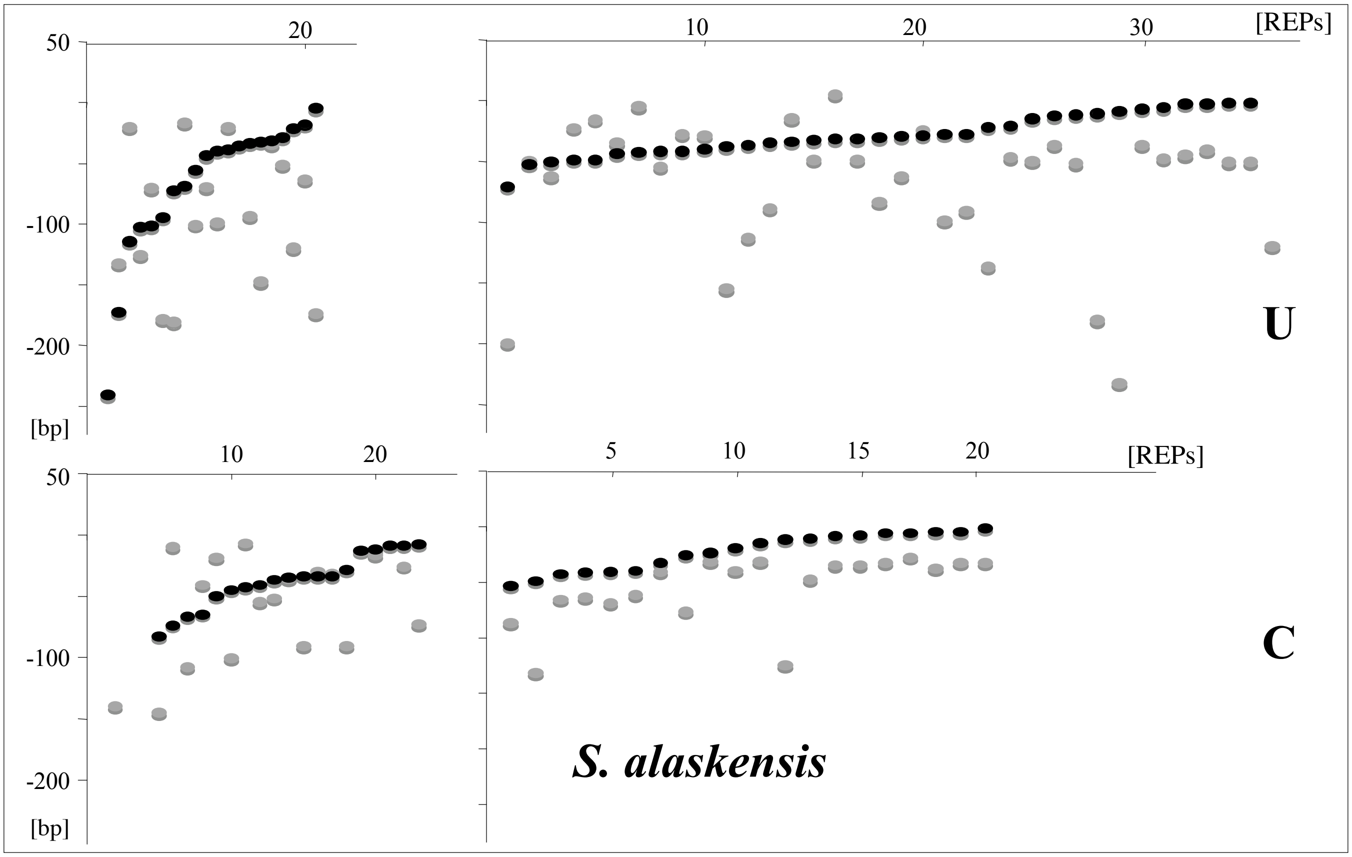
20
10
20
30
[REPs]
50
-100
-200
[bp]
U
10
20
5
10
15
20
[REPs]
50
-100
-200
[bp]
C
S. alaskensis
