# Supplementary material for: GTAG- and CGTC-tagged palindromic DNA repeats in prokaryotes
Source: BMC Genomics. 2013 Jul 31;14:522. doi: 10.1186/1471-2164-14-522 (PMC3733652; doi:10.1186/1471-2164-14-522)
Supplement: Additional file 5 — Alignment of RAYT and TIRYTs. Some of the identified RAYTs, and all the TIRYTs listed in Figure 9, have been aligned for comparison. [file 1471-2164-14-522-S5.doc]

**Additional file 5.** Alignment of RAYT and TIRYTs. The sequences of RAYTs (boxed) and TIRYTs are aligned. The conserved catalytic tyrosine and HUH domain are in bold face, motifs typical of RAYTs are in red, identities or favored amino acid substitutions, grouped according to Schwartz and Dayhoff (1978) in gray.

eco b228 MSEYRRYYIKGGTWFFTVNLRNRRSQ-LLTTQYQMLRHAIIKVKRDRPFEIN AWVVLPE **HMH** CIWTLPEGDDDFSSRWREIKKQFTHACGLKNI------WQPRFW EHAIRNT KDYRHHVD **Y**

sml 2816 MSSHRLRLGRHSIIGQSYVLTTTTHQRRRLFESEAAAACVIDQFHYIEQRGLVQSH AWVVMPD **HVH** WMFELRAAHLPDIARRMKSSSALALNRLVGRRCTV---WQSGYF DHAVRAE ESLAQQAL **Y**

sml 1101 MSRARLRLGRHSRIGQSYILTTVIQERRRYFDDATAAQEVMDVFRRIDAEGLTHSL AYVVMPD **HIH** WLVEIRAFSLDYIMQRFKSSSALRINRMLG-RTGPF--WQSSYH DHAIRSE ESLFRHAM **Y**

ote 2468 MLLPQRQTARLRTGRVSVPGATYFVTACVHHRAPVLTESRSATGLISTLSHLHERGDASII AATIMPD **HVH** LLFPLGGRLSLMQVVAKLKALSRRRHVSWE--------WQDNVF EHRLRAD EARDDYRF **Y**

rba11751 MHWSLVIQDRKQGWCKPILLYKFRELLTHTAFRYAIACP VYCLMPD **HIH** MLWMGLFESSDQRLAMRHFRDRFNDVLERLQVELQGQ-AYDDVL KDEERKE ESIEAVCD **Y**

rca 2775 MVLARARRSRLGRPQGVAPTITEPGSGRPQGVAQTIRQSQEGE PNLSLPD **VVH** RLKTMTTKLYADGVRQSGWPPFRSRL------------WQRNYH EHIIRDD NELSRIQQ **D**

pfs 3939 MAELPASHRLRIGRFSEPNRIYLITTNTHERIPIFSNFHLGRLLVWQFRLAQHQGLANSI AWVIMPD **HFH** WIIELKKGSLADLMRQVKSKSTRSVNGVSGRKGRL---WQESFH DRALRKE DDLVKMAR **Y**

pen 5170 MKIAHSHRLRTGRYSEPGRPYIITSVVLDREPLFSDFQLARLLVAELRHAHDQQWARSL WWVVMPD **HFH** WLAEPQEVDLSNLVRKVKSRSTRLINAAASRQGRL---WQKNFH DHALRQD EDIRTAAR **Y**

ppf 5083 MPLAESHRLRRGRFSEPGRLYMLTTITHQRKPLFRDFHHARLVVKHLRVSDDIQDCQSL AWVVMPD **HLH** WLIELKEVTLGTLMRKFKSRTAIALRKAGVGHKPI---WQSGYQ DHALRRE ECVVHVAR **Y**

ppf_607 MERPGSHRLRNGRVSESGRLYLLTTTTRSRTPLFKDFRFARTVIQQLRFSDERKACRSL AWVLMPD **HLH** WLIELGPV—-SLGRLMCEFKSRSSCALYKAGVERRHI-WQTSFH DRALRRE EDVRAVAR **Y**

pen_4846 MDSPCSHLLRRGRFSEPGRLYLLTTTTRDRKPLFADFNLARVVVKQMRLCDQKHACRTL AWVLMPD **HVH** WLVELGHARLSTLMCAFKSRSSNALYREGVERRHI---WQAGFH DRALHRE EDVKAVAR **Y**

pfs_4255 MRPTPQSHRLRHGRYSEHGRSYLITVVVHHRQRLFTDLSLGRLLVAEFRQAHERGLVDSL AWVIMPD **HIH** WLFELKQMTLADVVRRMKSRSTLTINRHRQSKERV---WQPGYH DRAVREE DDIRKMAR **Y**

aba 1552 MARPSRNADANAILSGARTFFVTTKTASGKRLLQSERNASLLIEVLRHYAREQKFEIH DFVVMPD **HVH** LLLTVDGETSIEKAVQFIKGGFSYRIKKDFGYPGDV--WQKGFS EVRVEDR ESYLRHRE **Y**

aba 3477 MPWGLKRYQHTGQSHFITFSCYHRLPYLSSTSAKQQFELSLEDTRVRYGMPVA GYVVMPE **HVH** LLVWEPQRSNLATALQSIKQSVSRLLIGGREHF-----WQKRYY DFNVRAA DKFWEKLH **Y**

tsa 392 MTRGLKRYQTSGHDHFLTFNCYGRRPYLSSDSACSLFEDALERMRIRYAFQVY GYVVMPE **HVH** LLVDEPKNGTLDEAIKGIKLSVTLRSRQRPF-------WLPRYH DFNIFTV TKFREKLR **Y**

aca 645 MPKALVRYQNTGDLHFVTFSCHGRKPYLDHASARELFEHSLEAMRQRYDFLLL GYVVMPE **HVH** LLVSEPREAMLAKALQAVKLSVAVQRRERPF-------WQARYY DFNVYSP AKRVEKLR **Y**

gma 794 MPTGLRRYQQAGDLHHITVSCVRHRPILGTPEARDIFLKLLERTREIYDMNVF GYVVMPT **HVH** MLVAEPNKAPLSVAMQILKQRFSKTRPEEDV-------WETRYY DFNVRTE AKRIEKLK **Y**

pfs 148A MARRPRVILPDVPLHIIQRGNNRSACFYSDEDYIFYVDKLDLLAELYGCKIH AMCLMTN **HVH** LLLTPTCFAGAGLLMKGLGQRYVQYINRTYQRT--GTLWEGRFR SCLVQKD NYVLACYR **Y**

sml 4509 MPRQARLMLAEQPYHVTQRGVNKGAIFVDDIDRQLFLHLLHSAFLKHQVALH AYVLMGN **HVH** LLATPSTRVGLANAMRMQGNNYVQAFNQRHQRS--GPLWQGRFH SSMVDSD AYLLSVYR **Y**

cps 4446 MARSPRLNLKDIPQHVIQRGNNRQSCFFENQNYQVYLDKLLEYSQKHQVEIH AFILMTN **HVH** LLVTPRIENGVSLMMQSLGRYYVRYINQTYHRT--GTLWEGRYK SSLVDSD SYFLIVTR **Y**

hch 1378 MARLPRLYLPGCAHHIIGRGNNREPCFYDEADYKAYLSFLKDAADKYQVDIH AFVLMTN **HVH** ILATPVDDQGISRMMQAQGRKYVQYFNYTHSRT--GTLWEGRYK STLVDSE TYLLTVYR **Y**

sse 3670 MPRAHRNSPVNIPQHIIQRGNNRQACFASEEDFMLYAKWLKQYASEYQVHIH AWVFMTN **HVH** ILCTPLQPNAISQMMQSLGRQYVRYFNFTYKRT--GTLWEGRFK SCLVQTE QYLLQVYR **Y**

swd 1484 MPRKPRFYAPDITCHIVQRGNNRSACFFNDDDFKIYINALSDALIQYNVKLH AFVLMTN **HVH** LLMTPSTTEGISQVMQSIGRTYVSTINRLYQRT--GTLWEGRHK ACLIDSE AYFIACLR **Y**

mpc 0880 MPRRPRVSIPGYAEHIIQRGNNRQPIFVSDEDIKAYAFWLGEYAKKFEVSIH AWVFMTN **HVH** LLCTPSNTTCVSKMMQSLGRQYVRYFNYTYQRT--GTLWEGRFK SCLVQDQ SYLFHLYP **Y**

psu 2716 MPRRPRLDLPGVSQHLIQRGNDRQPCFFADIDRVRYLHDLRELARTHDCQVH AYVLMTN **HVH** LLLSPARVRAVSALMQSLGRRYVRYFNDRYQRT--GTLWEGRYR SCLVDSE DYLLRCYR **Y**

tkm 1558 MPRMARVVLPHMPHHVVQRGHNRQVVFAEDGDYERYLEDLRELSSALDIRVY AYCLMTN **HVH** LLLGPGEEVAAMGRLMKALAARATRYRNRLEGR-SGTLWEGRYK SSPVQTE TYLLACTR **Y**

aba 2776 MARLPRVVAVGVPHHVTQRGNARQFLLTTDAERTIYLALLRQSAELRGLAVL GYCLMSN **HVH** LVVIPHHADSLALALKQAHGRYAAFWNASHQSS--GHVWQGRFF SCPLDRE EHLWRALR **Y**

bbt 5382 MARLARVVVPGLPHHVTQRGNGRARTFFGDDDYALYRDLLATHCQAADVAVW AWCLMPN **HVH** LILVPSDADGLRRALARVHRVYAGIIQARRKRR--GHFWQGRFG AVAMD-E AHLAAALR **Y**

ccr 1445 MARLARTVFEGVPHHVTGRGNGRAQTFFSDADFQLYRDLLAENAVKANVEVW GWVLMPN **HVH** LILVPSDPDGLRRCLAPTHRRYAGVIHAREKRT--GHFWQGRFG CVPMD-E AHLGAALR **Y**

mlo 6832 MARLARIVVPGLPHHVTQRGNGRAKVFFTPEDYALYNNLLVEHCRAANVGIW AWCLMPN **QVH** LILTPADLDGLRRALAKLHRAYAGIIHARQKKT--GHFWQGRFG AVAMD-E DHLLSAVR **Y**

gsu 2396 MARIARVIATGIPHHVTQRGNRRMPTFFNDEDYRAYIALLGEWCRKCGVDIW AYCLMPN **HVH** LIAVPETDDGLRRGIGEAHRRYSRMINFRENWR--GHLWQGRFA SFPMD-E SYLLAAAR **Y**

pca 3049 MPRIARIVAPGYPHHVTQRGNNRSTTFFDDEDRLRYLNQLKKYSTRHSLHIW AYCLMDN **HVH** LLVVPEKEDSLSRGIGLTNQVYTQYLNRKLKQS--GRVWQNRFF SCIVEDD AYLWRVAR **Y**

gbm 2474 MPRIARGLNDGFIYHVLNRGNSREEVFHKQGDYYSFVKLLTESMEQFDVRIF AYCLMPN **HFH** ILVEPEKGEALSKFMQWLTTCHVRRYHQHYGTS--GHVWQDRFK SFIVQND VHLLTVAR **Y**

dau 2153 MPRPVRGLADGFIYHILNRGNAKQVVFKKAQDYQVFVELMMEAKKRYSGIRLMAYCLMPN **HFH** LVLQPDQGIHLSQWMHWLMTTHVRRYHQHHQTG--GHLWQDRYK SFVIQDD DHLLTVLR **Y**

rba 2408 MPRAPRADASGHLYHVLNRANRRATIFRKQQDYEAFESVLAEALSKDEVELF SYCLMPN **HWH** LVLRPKCDGGMSRFVQWLTLTHTQRYNAHYEIVGEGHLYQGRYK SFPIQDD EHFLTVCR **Y**

sml 1152 MPRPRRVDAPGYPQHVVQRGNNRQPVFFTDGDHVAYLRLLCHHAHQQHCRVH AYVLMGN **HIH** LLATPDVCGGLSRMMQAVSRTYVRRVNERQGRT--GTLWEGRFH STVVDSD RYLLACQR **Y**

eco b228 IYI NPVKHGWVKQVSDWP FSTFHRDVARGLYPIDWAGDVTDFSAGERIIS

sml 2816 ILG NPVRAGLAGQIGEYP YAWSVWL

sml 1101 IVA NPVRANLASCVGEYP YAWCRWEIDERYQAMDYPEAER

ote 2468 IFM NPYAAKLIPTTACWP WWYCPEPSACRFLPLLHADGTPPREWLREREVIRNRLHVS

rba11751 IAR NPERAGLVPIDGYAD YKFTGCLVPGYPELTPFAADYWTRYWRIVSYLRSNGLQQGS

rca 2775 ILD NPARWDVDRESPAAV TPEPKCRREQ

pfs 3939 VVA NPLRAGLVKKIGDYP LWDAIWI

pen 5170 IVS NPLRSGLVRSLRHYP HWDAAWL

ppf 5083 IVA NPLRAGLVRSIRDYP HWDAVWL

ppf_607 IIA NPIQAGLVRRAGEYP HWDCVWL

pen_4846 IVA NPIRAGLAKRVGEYS HWDCVWL

pfs_4255 IIA NPLRAGLVERVGDYS LWDAAWL

aba 1552 IAK NPVNDGLVREGEEFA YCFTFLARQKAAGAKAPENIGAAVRQ

aba 3477 LHQ NPVKRGLVKSPEQWT WSSFRHHAFGEIGTVELESHWTALRRERAGIRFELKKDRDATTFGDPGSTKI

tsa 392 LHR NPVVRGLVSEPEEWG ASSYLHYLNGVRGRVEISSDWV

aca 645 MHR NPVKRGLVAQPEQWS WSSFRHYATGEAGTVEIQSSWTVNRRKKPPNPLS

gma 794 IHR NPVRDGLVKEPDEWL WSSFRSYRYLEPGPVTLSRP

pfs 148A IEM NPVRAGMVAHPGDYR WTSYRANAQGEPSSFLSPHAVYRELGSSTVQRAESYRSLFKDRLSPELIEQIRFSTNGNYVLGDQKFAAEIEQALGKRVSQGVSGRPRSVS

sml 4509 IEL NPVRAGMTAAPEDHP WSSVHGNLQRRHDPMLTEHPAFQALAQTQRQRGMLYAEFLQDMNASADLPAIRAHSAGQHPLGNAAYLRMVERTLGRPVMLRKRGRPQKKGAEG

cps 4446 IEF NPVRAEMVVRPELYP WSSYHQHALGKTISLLTEHDCYLKLGDSASKRQQSYLKFCKRYLSDDEASFIRASVNKAWILGDDRFKQQIERQLGVSVSPKARGGDRKSIKYQQSKG

hch 1378 IEL NPVRAEMVEHASEYP WSSYRHNALGRMIQLIRPHSIYQQLGKTAEERQKHYRLLFRGRMPEQDLSAIREATNKAWVLGNDRFKAEIEAKTGRRAKPLGRGGDRKSEKFRVMKDQ

sse 3670 IEL NPVRAEMVAEPSAYK WSSYQINALGKTSTLCTPHQTYLSLASNPTQRQINYRALFKHHIDTQLITQIRNATNKGMAIGNDKFKNEIEALTGESIRPKKRGRPSQK

swd 1484 IEL NPVRANMVKHPSEYP WSSYQFHGAGKQIACLTPHPLYQQLGSTPELRQHQYRELFNTELPPEQVHTIRNCLNHNFPLGSDKFRADIEAHLNVRFGHLAPGHPNKPKKT

mpc 0880 IEL NPVRADMVKDPADYS WSSYQCNGLGASSDLLTPHQLYQSLGRTKEERCNVYRDMFQYQVDGKLLEDIRLTANKGLALGNDKFKEQIELLTGQRQTEVKRGRKEGWRRK

psu 2716 IEL NPVRAGMVVDPADYP WSSHGANALGRPDPLVQPHPRYLALERDDDTRRAVYRSLVTRGLEPTEVEEIRRAMGTQRALGSDDFREDIQRRLGRRVAPGRPGRPRKQKPKPGSECNFPLESLRTPQASRHEIAL

tkm 1558 IEL NPVRARMVPAAGDYA WSSFRQRMGEEEQWIDLDPA-YLDLGHHEPDRRARYARFVEQGVPEPELTLVREALQRGQLTGNQRFVDEVEQIIGCRIERRRPGRPSSRRA

aba 2776 TEL NPVRAGLVLSAETWS WSSAGAHCGSTLRPGWLEMETWDSWWTPSAWREYLSAGETEGERI-----EIRRRTYSGRPLGEPAFVEQLEQAIHRQLTPRKRGRPAKEVPADHEESLISPE

bbt 5382 VSL NPVRARLVTRAQDWA WSSTRAHLRGRDDGVTAREPVKTMFPDMVGLLSRAPEDEEELFAR------LRAAESIGRPIGSDRFLTRIEKMTGRVLKPAKRGPKPAAED

ccr 1445 VAL NPVRARLVDRAEDWR WSSLHACLDPARGDGLTRTEPVLSRYPDFAAMLADGEDEAGSVA-------LRRAETIGRPVGEAVFLEDLERRSGRSLSPAKRGRKPKQN

mlo 6832 VGL NPVRAGLAKQATDWP WSSAQAHLTGEPDGVTDLQPMRDRLPSSSGLFDLVETDVAAFDA-------LRQAESIGRPVGDEVFLGQIAGQTGRALKPGKRGAAGQN

gsu 2396 VEM NPVRARLTPKAALWP WSSARAHLDRMDDELVKVAPLLELVGDWSLFLAGTGEEDQLND--------IRKHERTGRPLGTEEFVERLESALERPLKKGKPGPKGNDN

pca 3049 IEK NPLKAGMTNRAENYR WSSAKAHIKGSDEDLLAEYCWLPLEDRARYAEFMLQEDDQADNM-------LRKATCTGRPYGSEAFISKMELRLNKTLKAQKPGRPRKKTGECP

gbm 2474 IEG NPVRAGLVKSAKDWR WSSHRDRLKGA---------GNGTISDLPIALPDAWSSYTDLPLTSIELEKLRKSVAKRAPFGEEEWVHKTSEQHGLSSTLKGQGRPKKVMKIQS

dau 2153 VEG NPVRAGLVDFAEEWP WSSHRERVRGG----------RVLVDHTPVELPSNWKTYVDEPLTAKELETIRLSVVRRSPFGEPAWCNKTARELGLESTLKPRGRPRKHNR

rba 2408 VER NALAAGYCELPEDWR WGSLHRWKYGA-------AKEKALLSPWPIVRRSGWCQHVAAKLSDKEQKQLDFSVKRGAPFGEENWMESTARRFDLEMTMRPRGRPRKFEQRETTGYPVCQKGT

sml 1152 IEL NPVRAGKVAHPGDYR WSSYRANARGR-----------PNALLEPHSAFELMGSDADERRRRYVEFIEAGIPAGDLLAIRRALQSQRRLSGMLVGSEPFRIAKGL

**Reference**: Schwartz, RM, Dayhoff M0. In Atlas of Protein Sequence and Structure. Volume 5. Ed. Dayhoff, M. 0. Natd. Biomed. Res. Found., Washington, DC. 1978: 353-358.
